# Supplementary material for: Assessment of NUDT5 in Endometrial Carcinoma: Functional Insights, Prognostic and Therapeutic Implications
Source: Biomedicines. 2025 May 7;13(5):1136. doi: 10.3390/biomedicines13051136 (PMC12108576; doi:10.3390/biomedicines13051136)
Supplement: Supplementary file 1 [file biomedicines-13-01136-s001.zip › biomedicines-3507257-supplementary.pdf]

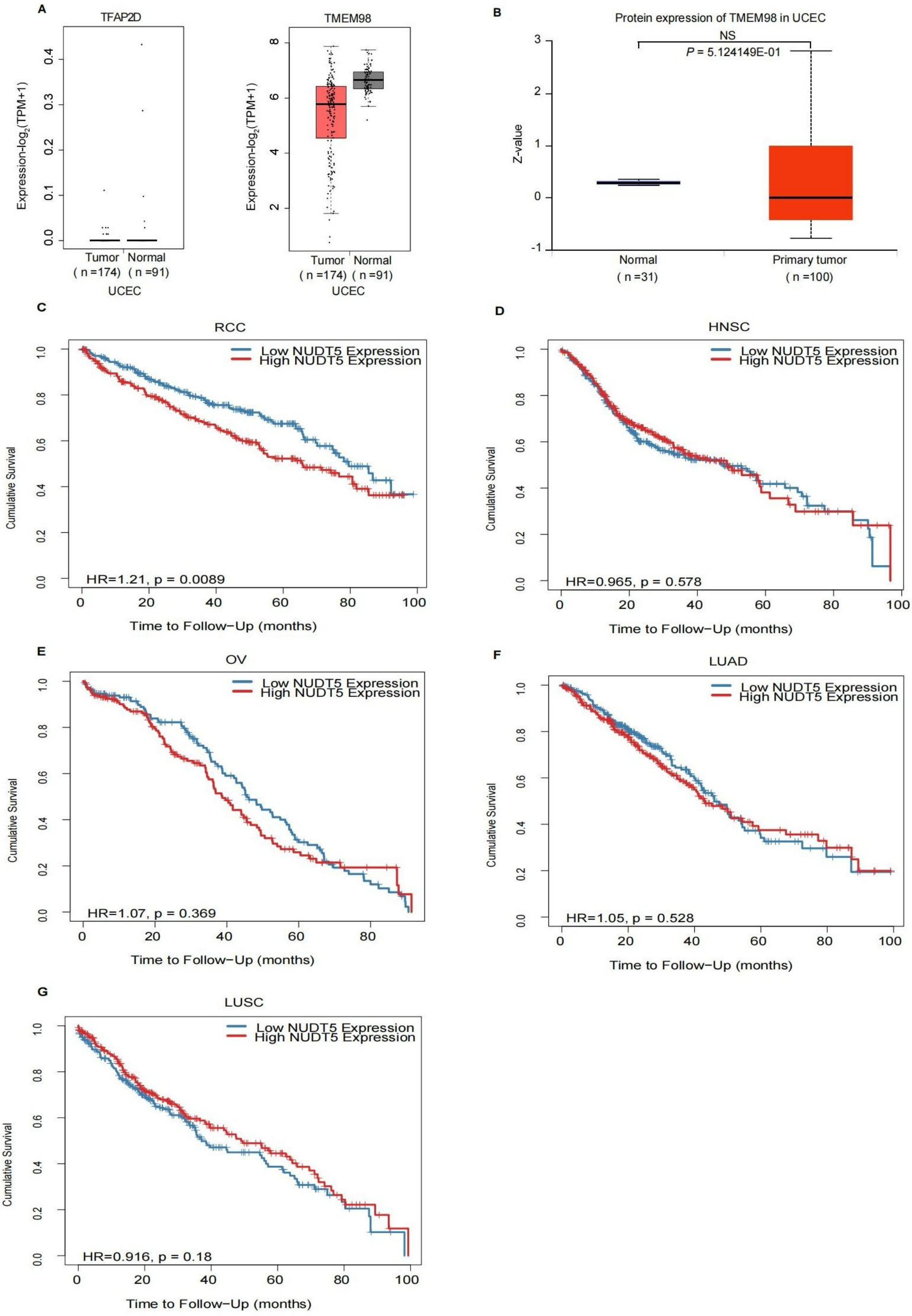

Figure S1. Identification of NUDT5 and its role in EC prognosis. (A) Analysis of TFAP2D indicates minimal expression in both normal and cancerous endometrial tissues. (B) Analysis of TMEM98 expression shows no significant

differential expression between normal and EC tissues. (C) Overall survival of patients with differentially expressed NUDT5 in renal cell carcinoma (RCC). (D) Overall survival of patients with differentially expressed NUDT5 in head and neck squamous cell carcinoma (HNSC). (E) Overall survival of patients with differentially expressed NUDT5 in ovarian cancer (OV). (F) Overall survival of patients with differentially expressed NUDT5 in lung adenocarcinoma (LUAD). (G) Overall survival of patients with differentially expressed NUDT5 in lung squamous cell carcinoma (LUSC).

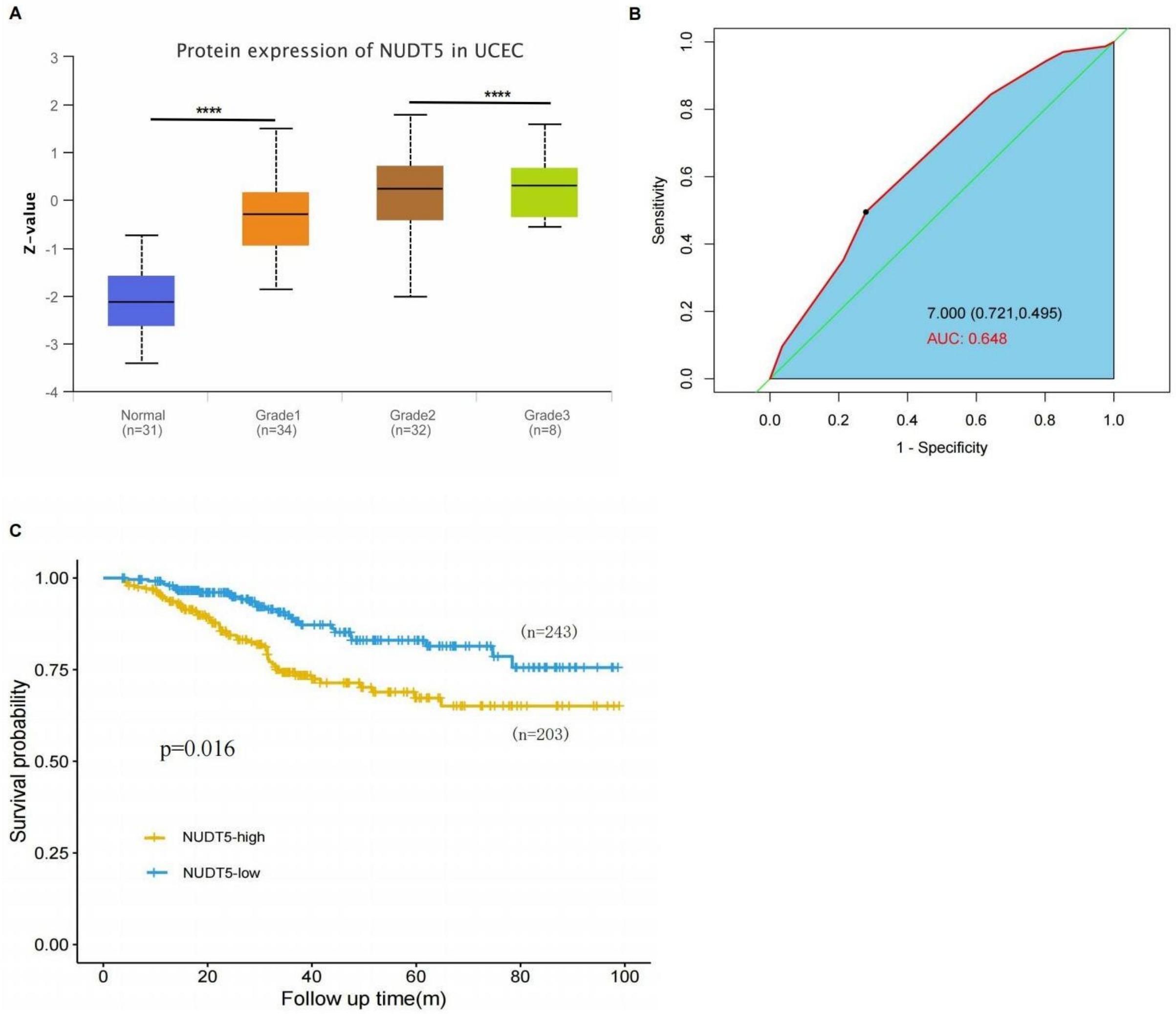

Figure S2. Identification of NUDT5 protein and its role in EC prognosis. (A) Positive correlation of NUDT5 protein with histological grading according to CPTAC. (B) ROC analysis for NUDT5 protein in distinguishing EC: optimal cutoff value of 7, AUC of 0.648, sensitivity of 0.721, and specificity of 0.495. (C) Higher NUDT5 protein expression predicts poorer prognosis in patients with EC.

Table S1. Clinical characteristics of EC and control groups and univariable/multivariable logistic regression for EC risk factors.

Factors	Levels	Control group(N=240)	EC group (N=276)	OR (univariable)	OR (multivariable)
Age		56.4 ± 9.8	57.1 ± 10.0	1.02 (1.00-1.04, p=0.096)	1.00 (0.97-1.03, p=0.974)
NUDT5		5.6 ± 2.5	7.0 ± 2.4	1.32 (1.20-1.45, p<0.001)	1.43 (1.27-1.60, p=0.011)

Factors	Levels	Control group(N=240)	EC group (N=276)	OR (univariable)	OR (multivariable)
Waist (cm)		85.7 ± 8.1	87.1 ± 8.7	1.58 (0.85-3.11, p=0.181)	1.46 (0.96-3.23, p=0.195)
Hip (cm)		104.6 ± 6.3	105.6 ± 8.3	1.09 (0.96-1.13, p=0.081)	0.85 (0.54-1.35, p=0.492)
BMI		24.0 ± 2.6	27.2 ± 4.2	1.62 (1.23-3.43, p<0.001)	1.55 (1.17-3.52, p<0.001)
WHR		78.5 ± 7.7	79.4 ± 7.1	1.02 (0.99-1.05, p=0.133)	1.12 (0.85-1.49, p=0.507)
Hypertension	NO	169 (82.8%)	173 (62.7%)		
	YES	35 (17.2%)	103 (37.3%)	1.93 (0.91-3.40, p=0.161)	3.08 (0.69-5.58, p=0.191)
Diabetes	NO	204 (91.5%)	198 (77.3%)		
	YES	19 (8.5%)	58 (22.7%)	1.80 (1.31-5.83, p<0.001)	3.84 (1.47-5.54, p=0.006)

Risk factors for EC occurrence were evaluated by univariate and multivariate logistic regression analyses, with results expressed as odds ratios (ORs) and 95% confidence intervals.
